# Stakeholder perceptions of India’s Digital Personal Data Protection Act of 2023: an empirical study across legal, banking, and corporate sectors

**DOI:** 10.3389/fsoc.2026.1753383

**Published:** 2026-05-29

**Authors:** Samreen Ahmed, Mohammad Nasir

**Affiliations:** Faculty of Law, Aligarh Muslim University, Aligarh, India

**Keywords:** awareness of privacy, data governance, data privacy, Digital Personal Data Protection Act of 2023, informational privacy, stakeholder perceptions

## Abstract

The Digital Personal Data Protection Act, 2023 marks a watershed in the India’s data protection regime, building on Supreme Court’s recognition of right to privacy including informational privacy, as a fundamental right. As India’s first cross-sectoral law on privacy, the Act has come under close scrutiny amidst the proliferation of the data-intensive technologies. Drawing on a survey from 380 respondents—lawyers, employees of corporations, bank employees, and laypersons, the paper employs IBM SPSS, to examine the perception of Act’s impact on ease of business and innovation; its effectiveness in protecting data privacy, its capacity to balance privacy with business needs; adequacy of its grievance redressal mechanism, and levels of rights-awareness. The findings reveal sectoral variations in the understanding, expectations and compliance preparedness. The paper argues that, with the Act and its Rules awaiting enforcement, aligning legislative intent with stakeholder experiences is crucial to ensure practical efficacy in India’s digital economy.

## Introduction

The foundation of the India’s data protection regime was laid down by the Supreme Court’s decision in *Puttaswamy* (9 judge bench decision) (Justice, 2017), where the right to privacy was recognised as a fundamental right and informational privacy as its subset. The court observed that the contemporary information age, the dangers to privacy may emanate from state as well as non-state actors. Accordingly, it commended the Government of India to put in place a carefully structured data protection regime that is sensitive to the individual interests and legitimate state concerns. The court also took note of the existence of Justice BN Srikrishna Committee which was entrusted with the task of suggesting a data protection framework for India, and ruled that any resultant regime must take note of the judgement and the principles laid therein. The judgement marked a shift in Indian privacy jurisprudence from a narrow approach focusing solely on privacy harms (resulting from violation) to a broader conception wherein privacy is viewed “as a right worth protecting in itself” (Burman, 2023).

Recognising that the right to privacy, like other fundamental rights, is not an absolute right, the Apex Court laid down a test on the anvil of which any law impinging upon the privacy right has to be examined. The three-fold requirements of the test include “(i) legality, which postulates the existence of law; (ii) need, which is characterised by of a legitimate State aim; and (iii) proportionality which ensures a rational nexus between the objects and the means adopted to achieve them.” This test continues to be the benchmark for evaluating the data protection laws in India, including the recently enacted Digital Personal Data Protection Act, 2023 (DPDP Act, 2023).

Against this backdrop, this paper draws on empirical data collected from lawyers, bank employees, and corporate sector professionals to examine perceptions about the DPDP Act’s effectiveness and its implications. It investigates four interrelated themes: (i) awareness of rights under the DPDP Act; (ii) perceived effectiveness of the Act in protecting personal data; (iii) assessment of the impact of government exemptions on the corporations’ operations (iv) views on Act’s potential impact on ease of doing business, innovation; and (v) opinions on the Act’s ability to balance privacy protections with the needs of business and data processing. The paper contributes to the ongoing law and policy debate relating to data privacy and protection, by examining through an empirical study, how the professionals across sectors understand and evaluate the India’s current data protection regime. In doing so, the paper identifies the gaps that exist between the legal architecture and perception of professionals, and assesses how far the DPDP Act withstands the constitutional standards spelled out in the *Puttaswamy* judgement.

### The need for an empirical enquiry

India’s legislative journey of data protection law has been shaped by a complex interplay of constitutional mandates (Justice, 2017)^1^, expert recommendations, business interests and political negotiations. Following the recognition of privacy as a fundamental right by the Apex Court in *Justice K. S. Puttaswamy v. Union of India* (2017), a committee headed by Justice B. N. Srikrishna released a comprehensive report in 2018. The report was accompanied by a draught Data Protection Bill. This was followed by a series of iterations, including extensive revisions by the government and a close scrutiny by the Joint Parliamentary Committee. Each version of the Bill reflected shifting institutional priorities and tensions between individual rights and state or business interests. After a delay of approximately 6 years, the DPDP Act was finally enacted in 2023, reflecting a landmark development. However, simultaneously it raised concerns pertaining to transparency, accountability, and the narrowing scope of protection. Considering the normative weight of the *Puttaswamy* (9 judge-bench judgement) and the expansive legislative re-drafting process, it becomes crucial to empirically examine how the DPDP Act is understood by stakeholders, who are most impacted by the Act’s provisions. An in-depth investigation into the perceptions and opinions of professionals across banking, legal and corporate sector provides valuable insights into how well this new law aligns with the practical demands and constitutional mandates. This forms the rationale of this study.

### Objectives of the study

This study seeks to examine how the professional stakeholders, namely lawyers, bank employees, and the employees of corporations perceive the provisions of the DPDP Act, 2023. It investigates their awareness of the rights under the Act, assesses Act’s effectiveness in protecting the personal data, analyses views on its ability to foster innovation, enhance the ease of doing business, and the ability to balance privacy/data protection with the operational needs of the data driven economy. By gauging the stakeholder perspectives, the study seeks to examine whether the DPDP framework aligns with constitutional expectations and the practical demands and realities of the economy.

## Research methodology

This paper adopts both—doctrinal and empirical methods for a comprehensive analysis of the Digital Personal Data Protection Act of 2023. Initially, the scholarship on the right to privacy and data protection available in form of research papers, working papers and book chapters was systematically reviewed to identify how the privacy jurisprudence originated and shaped the discourse in India with respect to data protection. The reports of consultancy and research organisations like Carnegie, Vidhi Centre for Legal Policy and PRS Legislative were perused to identify the various considerations of the government that formed the legislative intent behind the Digital Personal Data Protection Act of 2023, stance on the right to privacy, exclusion and inclusion criteria for data protection, and the lacunae in the proposed and enacted legal framework. This informed the theoretical underpinnings of the study and the questionnaire for the empirical part.

The questionnaire for this study was developed in consultation with statistical and legal experts and divided into two sections. Section 1 contained general questions pertaining to privacy and data privacy, whilst Section 2 focused specifically on the provisions, perceived implications and effectiveness of the DPDP Act, 2023. The total sample of population comprised 380 respondents, which included both laypersons and professionals. The professional group, containing 213 respondents, was further categorised into three subgroups: employees of corporations (also written as corporate employees), lawyers, and bank employees. This subgroup comprised 89 employees of corporations, 65 lawyers, and 59 bank employees. Laypersons were asked to respond to only first section of the questionnaire, whilst the professional group was given both sections. Data collection was carried out using a mixed-method. Some responses were collected via printed questionnaires, whereas majority were gathered through Google Forms. This technique ensures accessibility and ease of response.

A mixed sampling strategy was employed for the study to suit the heterogeneous nature of the respondent groups and the absence of a unified sampling frame. Layperson’s data was collected through simple random sampling across India, with a deliberate focus on the educated population, for eliciting informed perceptions of privacy and the related rights in the contemporary data driven environment.

For the professionals, a purposive stratified random sampling strategy was adopted. The first step involved identification of the three analytically relevant strata—employees of corporations, bank employees and lawyers—on the basis of their regular engagement (functional proximity) to the interpretation, implementation and compliance requirements of the DPDP Act, 2023.

At the second stage, for each stratum, respondents were selected through random sampling. This enabled a systematic comparison across professional groups whilst simultaneously minimising selection bias. This combined approach reduced bias, facilitated the data collection from stakeholders who were directly relevant to the study’s objectives and also facilitated a comparative analysis of privacy awareness and perceptions of the Act.

The sample selection for this study was guided by doctrinal relevance as well as the functional proximity to the operation of the DPDP Act, 2023. Lawyers were included in the sample because they directly engage with the DPDP Act in advisory, compliance and in the adjudication process. Moreover, their understanding reflects the legal and constitutional articulation of privacy and data protection.

Since, the corporate and banking sector are most data intensive sectors. The bank employees and employees of corporations were selected not as data fiduciaries in their individual capacity, but as operational representatives of institutional data fiduciaries. They are directly involved in handling and processing of large volumes of sensitive personal and financial data of individuals, and therefore bear the major compliance burden under the Act. The issues pertaining to consent management, data governance, cross border processing of data and other regulatory compliances are regularly dealt by them.

The perspective of data principals was captured through the laypersons, whose awareness of the privacy and related rights under the Act and remedies was crucial in evaluating the effectiveness of the Act in their estimation and its understanding beyond professional setting.

Together, these groups represent the key stakeholders who are engaged with the DPDP Act from the regulatory, operational as well as rights-holder perspectives, expanding the scope of the study beyond institutional and regulatory perspectives. This selection also enables a comparative assessment of privacy awareness and perception of the Act across those who interpret the law and those who implement/comply with it, those who are governed by it, and the data principals (those whose rights it seeks to protect).

Further, this dual perspective helped us situate professional insights within the wider contexts of public understanding, thereby enhancing the depth and relevance of the analysis.

The responses gathered were compiled and analysed using IBM SPSS Statistics 25. The statistical tests were employed based on the structure and measurement level of the data:

- The Chi-square test was used to examine the association between the two categorical variables, for instance, professional background and opinion with respect to DPDP Act.
- The *t*-test was employed to compare the means of two independent groups on interval-level variables.
- Kendall’s tau test was used to measure the ordinal associations between ranked variables, such as agreement levels on Likert scale.

These statistical tools and techniques were chosen to ensure that ordinal and nominal data could be analysed appropriately, adhering to the best practises of research. Most questions in the questionnaire were articulated as statements, and respondents were asked to indicate agreement level on a five-point Likert scale, ranging from strongly agree to strongly disagree.

### Theoretical underpinnings

#### Understanding privacy

Understanding privacy and its dimensions is crucial for evaluating the legal frameworks pertaining to data protection such as the DPDP Act, 2023.

Privacy is a multi-dimensional concept. Scholars broadly agree that it cannot be reduced merely to a singular definition or idea such as ‘right to be let alone’ (van der Sloot, 2021), rather, it encompasses a range of interrelated facets. For instance, in the contemporary era of information technology and artificial intelligence, ‘informational’ privacy has gained significance and is taking the center stage. Warner and Stone aptly describe privacy as a highly slippery virtue—intangible, hard to define and measure (Warner and Stone, 1970). Despite such elusiveness, time and again scholars have attempted to define it from diverse perspectives.

Contrary to the popular perception, privacy is neither limited to concealment of information, nor to personal autonomy or freedom (Prosser, 1960). The legal discourse on the ‘right to privacy’ originated with Warren and Brandies’s seminal article for the *Harvard Law Review,* wherein they argued for its recognition as a separate cause of action in tort law (Warren and Brandeis, 1890). Subsequently, Judge Thomas Cooley observed that privacy meant the “right to be let alone,” adding further legitimacy to the concept. Building on these early moorings of privacy, Gavison (1980) observed that rampantly emerging novel threats to privacy, along with their interrelationship with allied values like liberty, mental peace, have propelled most nation-states to recognise the right to privacy.

The scholarship on privacy can be broadly categorised under three approaches—structuralist, individualistic, and integrative. Structuralist scholars define privacy in relation to social structure and institutional frameworks (moral and legal rights) which operate to restrict others from accessing their information. For instance, Anita Allen, a noted academic characterises privacy as denoting “a degree of inaccessibility of persons, their mental status, and information about them to the senses and surveillance devices of others” (Allen, 1992). Similarly, Hyman Gross views privacy as control over access to one’s personal affairs, Gross (1971) whilst Bok (1982) ascribes privacy to secrecy, describing it as “a condition of being protected from unwanted access by others.” The structuralist approach in equating privacy to secrecy, restrict the reach of external scrutiny.

The individualists emphasise control over personal information. They consider privacy as a control measure—how much personal information we can keep with us and not divulge to others. American Professor Jeffrey Rosen notes that Alan Westin transformed this discourse by defining privacy as the ability to control how much about ourselves is disclosed to others (Fox and Westin, 2013). Westin defined it as “the claim of individuals, groups, or institutions to determine for themselves when, how, and to what extent information about them is communicated to others” (Westin, 1968). Similarly, DeCew perceives privacy as a protected space where individuals can shape their lives and relationship without fear of scrutiny, intrusion or judgement (DeCew, 1997).

The integrationists combine both structuralist and individualist frameworks, conceptualising privacy both as a right and an interest that serves broader societal purposes. They view privacy not as an isolated entitlement but something that adds value to the society (Koops et al., 2017). Reimen underscores privacy as “an important interest in simply being able to restrict information about, and observation of myself regardless of what may be done with that information or the result of that observations” (Reimen, 1976). In a similar vein, Justice Steven of the United States observed, “both the common law and the literal understandings of privacy encompass the individual’s control of information concerning his or her person” (United States Department of Justice, 1989).

These perspectives underscore that privacy is a nascent, evolving and layered concept, shaped by legal, philosophical and technological developments.

Although data protection and privacy are interrelated and former emanates from the informational privacy dimension. Noted author and academic, David H Flaherty notes that often privacy and data protection are conflated (Flaherty, 1991). He explains that ‘data protection’ is primarily concerned with the regulation of how one’s personal information is collected, used, processed, and shared. This is often done through laws that do not define or mention the word “privacy,” although the aim of such laws is to protect privacy. These laws operationalise privacy protection through fair information practises. Flaherty notes that this arrangement is better than featuring the word ‘privacy’ in statutes without even defining it. The DPDP Act, 2023, exemplifies such a model. Whilst intending to protect informational privacy, the Act does not define privacy, rooting it in constitutional philosophy. Rather, it emphasises on regulating the data processing, conduct of data fiduciaries and processors, and outlines the conditions for lawful processing. The Act illustrates a functional approach to privacy by emphasising on roles, responsibilities and procedures. It relies on delegated Rule making and compliance mechanism. This Indian law is a contemporary example of Flaherty’s insight that modern legal systems often tend to pursue data protection through technocratic regulation, whilst keeping the normative aspects of privacy undefined, perhaps, deferred.

#### Overview of the key provisions of the DPDP Act of 2023

The DPDP Act, 2023 marks a watershed moment in the data protection regime of India. Nonetheless, it is surrounded by criticism owing to its inherent weaknesses and delay in enforcement. It received the President’s assent in August 2023, after being rushed through the Parliament, passed with a week—without any substantial debate. This expeditious legislative process followed several iterations of the proposed data protection Bill, including the draught law proposed by the Justice BN Srikrishna Committee, alongside its Report (Ministry of Electronics and Information Technology, 2018). The framework suggested by the Committee was aligned with the International Standards (Kumar, 2022). However, the numerous Government re-draughts and recommendations of the Joint Parliamentary Committee (2021) ruined the Bill of all its strengths. They progressively weakened the final Act instead of strengthening it.

The final Act passed after 5 years of the delivery of Srikrishna Committee Report, appears to be more of a new draught that a redraft of any previous iteration and has inherited many weaknesses. A major concern is the narrowing scope of the protective ambit and wide exemptions afforded to the State. For instance, the Act applies only to the processing of ‘digital personal data’—data which is collected in digital format or has been subsequently digitised (Section 3(a)). This limits the remit of law, particularly with respect to the paper-based transactions or data noted paper, which is not digitised. The non-personal data records such as business analysis and anonymised data also are beyond the scope of this Act (Gupta and Naithani, 2023).

Despite the long gestation period, the provisions of the DPDP Act are not reasonably manoeuvred. They raise critical concerns about its effectiveness as a data protection framework for a rampantly growing economy of India, which is expected to have 1.08 billion users by 2026 (Sun, 2024). The DPDP Act adopts a conventional and narrow approach in defining ‘personal data’, further raising concerns about the effectiveness of the Act. Section 2(t) defines personal data as, “data about an individual who is identifiable by or in relation to such data.” The individuals referred here are ‘data principals’ (data subjects) who can control how their personal data is used by data fiduciaries (data controllers) often along with data processors who are hired through a contractual arrangement. Arguably the term ‘in relation to’ used in the definition requires a broad interpretation encompassing the content, purpose and the consequences of data processing (S&R Law, 2023).

Two major shortcoming that emerge are: the exclusionary effect of the definition and the absence of classification of data.

Featuring an abruptly sharp departure from the previous iterations and global standards, the DPDP Act, 2023 does not categorises the data. It treats at par all kinds of personal data. This is in stark contrast with the international practises, where sensitive personal data such as, “race, caste or tribe, criminal records, religious or political beliefs, sexual orientation, health, financial affairs, biometrics, genetic characteristic or any other characteristics,” are afforded greater level of protections. Noted academician Graham Greenleaf notes under the data protection laws, special safeguards are prescribed for handling such categories of data (Greenleaf, 2023). Even, the earlier draught Bills of Indian data protection law contained protective clauses for such ‘sensitive personal data’ including these kinds of data, except race and criminal records. Similarly, the Information Technology Act, 2000, also differentiated between the sensitive personal data and personal data. Rule 3 of SPDI Rules define ‘sensitive personal data or information’ (SPDI) as consisting of information relating to items such as passwords, financial information (e.g., details related to a bank account, credit/debit card or other payment instruments), health and medical records, biometric information, *et cetera.*^2^

The other weakness is that the definition of ‘personal data’ is so worded that it operates to exclude any data which the data principal makes publicly available or is so made available by any other persons is exercise of any legal obligation (section 3(c)). The General Data Protection Regulation (GDPR) of the European Union (EU) on which the Indian law is modelled, also along with other countries treats such publicly shared information as personal data. India, in this case, seems to have adopted the limited approach like Australia and Singapore. Arguably, with the burgeoning social media industry, such exclusion may adversely impact the data principals, as individuals casually share their personal data on social media platforms.

The definition of personal data under DPDP Act lacks the protective depth and the layering which was proposed in the earlier iterations and is available in laws of other countries, thereby weaking the legal shield it seeks to offer and the growing challenges of the contemporary society.

#### Exclusion of anonymised data

Anonymization is a commonly used practise that involves removing identifiers from personal data to reduce the risk of re-identification of the data principal. The DPDP Act and the Draught Rules are currently silent on whether anonymised data falls outside the purview of the legislation. Further, neither the Act nor the Draught Rules define anonymization or envisage any legal standards for anonymization. This assumes significance given the growing evidence that it may be possible to identify individuals from datasets that appear anonymised, particularly when combined with other datasets.

Currently, many data fiduciaries use anonymization techniques to process personal data for research and development. Unlike other countries and the PDP Bill 2018, the absence of a clear definition of “anonymization” and a framework for anonymization standards under the DPDP Act creates legal uncertainties. This is particularly concerning given the broad definition of “processing” under the DPDP Act, where any automated operation on personal data (which can include anonymization) qualifies as processing. Without clear guidance, data fiduciaries may adopt varying anonymization techniques, potentially using them as a loophole to evade compliance with the DPDP Act. Establishing robust anonymization standards is essential to ensure data privacy whilst enabling responsible research and innovation.

#### Automated and notified exemptions for the government

The DPDP Act in extending wide exemptions to the ‘State’ (Section 2(zb)) adopts the definition of ‘State’ under Article 12 of the Constitution of India. Article 12 defines State as “The State includes the Government and Parliament of India and the Government and the Legislature of each of the States and all local or other authorities within the territory of India or under the control of the Government of India.”

Within this architecture, the Central government, primarily, enjoys the exemptions. The Act defines ‘exemptions’ by using specific legal terms such as ‘specified’, ‘prescribed’, and ‘notified’—each of which carries a different degree of Parliamentary oversight. For instance, the exemptions created by government delegation that are ‘specified’ are subject to Parliamentary disallowance. The delegations pertaining to matters that are ‘prescribed’ are required to be tabled in the Parliament but are not disallowable, whilst matters that are ‘notified’ are not disallowable. The ‘prescribed’ exemptions give greater discretion to the executive.

Under the scheme of the DPDP Act, the exemptions shall operate as automatic exemptions and notified exemptions. For instance, Section 17 (4) automatically exempts the State and its instrumentalities from the obligation of data erasure on request or automatically as a legal mandate under Section 8(7) and Section 12(3). Similarly, it is exempted from the obligation to correct personal data upon the request of the data principal. Additionally, various automatic exemptions operate to cover processing of data for enforcement of legal rights, judicial functions, regulatory functions, enforcement of law, and in cases of credit defaults, and company reorganisations, is exempted under various provisions of the Act.

In effect, the notified and the automatic exemptions operate to permit the government and its agencies (wherever mentioned) to collect and accumulate the personal data without consent of the data principles, retain it for longer durations, override the data deletion requests and disregard the obligation of ensuring data accuracy.

This framework is criticised for paving way for profiling of individuals and for pervasive surveillance.

#### Processing for legitimate purposes

The DPDP permits non-consensual processing of personal data under certain conditions labelled as ‘legitimate uses.’ Such processing is also complemented by the Draught DPDP Rules. However, the scope of these ‘legitimate uses’ for which personal data is allowed to be processed without consent is significantly broad. This raise concerns over the lack of mandate of notification to the data principal of the use of their data and the absence of any required nexus or connection between this ‘new’ use and the ‘original use’/ ‘purpose’ for which the data was initially given.

Section 7(a)^3^ allows personal data of an individual to be processed without consent when the data principal has voluntarily provided her personal data for a specified purpose. This provision is distinct from situations where personal data is provided pursuant to the data fiduciary’s request. The notice and compliance requirements under sections 5 and 6 do not apply in the case of section 7(a) (Vaitheeswaran, 2025). Critics perceive that this distinction undermines the uniform protection of personal data, making it susceptible to privacy breaches.

Section 7(b)^4^ enables non-consensual processing by State or its instrumentalities for provision of subsidy benefits, service, certificates, licences or permits. The grounds and standards for processing personal data by the State for these purposes found in the Draught Rules (Rule 5 r/w Second Schedule) read with section 7(b), have garnered criticism for not fulfilling parameters prescribed in the judgement in *KS Puttaswamy v Union of India* (Justice, 2017). The judgement mandated that that any state action infringing upon the right to privacy must be backed by law. The reliance on policy-based authorizations for exemptions under section 7(b), fall short of the scrutiny prescribed by the judgement.

Moreover, section 7(c) allows non-consensual processing for government functions linked to the sovereignty and integrity of India, national security and friendly relations with foreign States. It also empowers the Central Government to exempt any instrumentality of the State from the provisions of the Act (Abraham, 2025). Arguably, public sector companies, law enforcement agencies, or anybody or entity carrying out State or government functions or operating under government control could be classified as such ‘instrumentality,’ and such exemptions could potentially extend to them. The Justice B. N. Srikrishna (retd.) cautions that lack of a definition for ‘instrumentality’ may lead to expansive and unchecked access to personal data of individuals. For instance, any bureaucrat or government department’s clerk may ask for data access for a matter pertaining to the sovereignty of India (Suraksha, 2022).

Section 7(d) to (h) further expand non-consensual processing of personal data for an array of situations—including legal compliance, compliance with the judgement/decree/order of the law courts, attending to medical emergencies or responding to threats to public health, and disasters. Although, such exemptions may be justified on the ground of public interest, the extent of permissible use warrants caution against potential overreach.

Section 7(i) introduces a more contentious exemption by allowing employers to process employee’s data without consent for legitimate purposes, vaguely categorised as ‘purposes necessary for employment’. The provision is open-ended and does not clarify what constitutes sufficient ‘relatedness to employment.’ Considering the sensitivity of the data, these purposes should have been illustrated as a list. Moreover, the examples listed under the section such as “*corporate espionage, maintenance of confidentiality of trade secrets, intellectual property, classified information or provision of any service or benefit*,” reflect corporate interests rather than privacy safeguards. Ravindran and colleagues from Vidhi Centre for Legal Policy contend that these purposes could be taken as valid grounds for prosecution of a person in case of violation of statutory or contractual rules on confidentiality but cannot serve as valid grounds for overriding an individual’s privacy. They suggest deletion of the purposes pertaining to confidentiality and IPRs. Arguably, closed list of narrowly defined employment-related purposes would have been appropriate (Ravindran et al., 2023).

For safeguarding data, non-consensual processing should be sparingly allowed, restricted only to cases where obtaining an employee’s consent is not feasible or appropriate. The Act should furnish data principal, the choice to opt out of such processing. Arguably, the bargaining power disparity between employers and employees warrants legal protections for the less advantaged, i.e., employees. For instance, an airline stewardess may be asked to furnish her children’s and her parents’ personal details. Children’s data can be taken for legitimate use for employment benefits, but collecting parents’ data by the airline does not appear legitimate, where they are not dependants. Such data collection under the pretext of employment benefits highlights the coercive potential of such broad exemptions.

Thus, the drafting of section 7 of the Act thwarts its attempt to enable pragmatic data processing. The lack of clarity and adequate cheques coupled with the absence of proportionality raises several concerns, *inter alia,* of potential misuse and weakened data protection safeguards.

The Rule 15 of the Draught DPDP Rules exempts the application of the DPDP Act in the cases where the processing of personal data is essential for research, archiving or statistical purposes., subject to the standards prescribed in the Schedule Second. It is yet to be clarified whether ‘research’ includes ‘commercial research’ and research conducted for technological advancements, like training the Artificial Intelligence (AI) models (Chaudhuri, 2025). The law has to balance the need for regulation of data against the need for innovation. AI models need access to datasets for training as well as for learning (Shekar and Sharma, 2025). As India strives towards create an enabling environment for AI innovation, ensuring access to datasets for technological research will be crucial. However, the standards applicable to research exemption as proposed by the Draught Rules will have to be amended to include reasonable conditions.

#### Unfettered power to requisition data from fiduciaries and intermediaries

Rule 22^5^ of the proposed DPDP Rules grants the union government the power to compel the disclosure of personal data. Specifically, it empowers the government to require any data fiduciary or intermediary to provide information for the purposes mentioned in the Seventh Schedule of the DPDP Act, within a prescribed time frame. The only exception carved out is where such disclosure affects the sovereignty and integrity of India, a restriction that may be waived with prior written permission from the person authorised by the central government. Legal scholars and privacy experts perceive this provision as conferring arbitrary powers in the government. They argue that it opens vistas for unchecked data collection and mass surveillance. Further, absence of any recourse mechanism for data fiduciaries and data principals increases the risks of unjustified or excessive data collection, resulting from misuse of power under Rule 22 read with Section 36 of the Act.

This reflects the broader trend in the architecture of DPDP law of power concentration in the hands of executive, often at the cost institutional accountability and the privacy rights of individuals.

#### Rights of data principals

Section 12 of the DPDP Act extends to the data principles three core rights aimed at enhancing autonomy of the data principal^6^. These rights include the “right to access personal data,” right to erasure or correction of personal data, withdraw consent, and a right to nominate a representative to exercise these rights on their behalf (Singh and Anusha, 2024). Collectively, in effect, they operationalise the right to informational privacy.

1 The right to access: The data principal is vested with the right to access their data held by a data fiduciary. This includes information regarding the source of the data, purpose for which it is processed, and third parties with whom it has been shared. For example, if the email address of a person has been processed, the data fiduciary shall have to disclose how and from whom it was obtained and to whom it has been disclosed.
2 The right of correction. The Act entitles a data principal to request for correction, completion, or updating of data. A practical example could be a woman seeking to update her surname after marriage, the data fiduciary would be obligated to update this change, upon her request.
3 The right of erasure: A data principal can get their personal data deleted. This right can be exercised only in two cases, where the data is no longer required or the data principal has withdrawn their consent. However, this is subject to two limitations—data may be retained if required for a specified lawful purpose or for compliance with any law for the time being in force.
4 Right to withdraw consent: A data principal has the right to withdraw their consent at any point of time. In other jurisdiction, this right, in general, is exercised through consent managers who offer user-controlled preference centres for revoking consent.

Other than the rights contained in Section 12, the Act recognises some additional rights, including the right to object to or restrict data processing, and a limited right to data portability. These provisions attempt to align Indian data privacy framework with the global standards.

##### Right to data portability

‘Data portability’ refers to the facility of obtaining a copy of one’s personal data in a readable format and transfer it anywhere. For example, health records of a person can be moved from one doctor to another. Under the DPDP Act, there is limited data portability that if facilitated through the consent managers who act as a medium between the data (user) and the platforms. The Act uses the term “interoperable platform” in defining consent managers, this reflects the legislative intent to make consent managers as facilitators of data portability. These consent managers are registered with the DPB. The Act envisages them as contact points for users for various functions—giving, managing, reviewing and withdrawing their consent (Vrabec et al., 2024).

#### Stakeholder engagement and compliance challenges

India’s data protection law—DPDP Act, 2023, adopts the same structural trifecta as the General Data Protection Regulations of the European Union, however, with a slightly modified nomenclature—data principal (data subject), data processor and the data fiduciary (data controller). Whilst, the GDPR is prescriptive, the DPDP Act defers most of the thornier aspects to the delegated legislation. As many as 28 times, the Act has employed the phrase ‘as may be prescribed’, leaving key substantive and procedural aspects for the Rules to cover.

Such a legislative technique has been criticised by scholars and practitioners. Graham Greenleaf argues that such excessive delegation of powers to the executive without corresponding cheque and balance mechanism in the parent Act, makes much room for misuse of authority.

Although, the DPDP Act has borrowed much from the EU’s GDPR, and strives to align with the international standards, its over-reliance on the discretion of the executive by leaving much for the Rules to prescribe, dilutes the clarity, enforceability and effectiveness of the privacy protections.

Privacy law expert, Sarah Abrham views the Act and the proposed rules as manoeuvred in a ‘bare-bones model’ fleshing out only the fundamental principles of data privacy. She finds the framework as gravitating towards the consent. She contends that the novelty of the Indian privacy regime and the paradigm shift in the mechanism of dealing with personal data, has left most of the stakeholders in a state of confusion, prompting them to adopt a cautious approach of wait and watch.

The excerpts and media reports published after the publication of the DPDP Act in the Official Gazette reported that a sense of panic was witnessed in companies. They were concerned about the timelines stipulated under the Act. Many expressed uneasiness about the feasibility of implementing the procedural and technical safeguards within the specified timeframes. However, with the delay of approximately 2 years in notifying the date of enforcement and the persisting uncertainty about its operationalisation, these concerns appear to have receded from the public discourse, albeit temporarily. The absence of any official communication from the government regarding the date of enforcement of the Act has muted the earlier apprehensions, nonetheless, the compliance challenges remain unresolved.

This shift indicates that whilst initial panic was triggered by statutory timelines, prolonged legal and regulatory ambiguity has created a different sort of uncertainty, one marked by institutional pause than by preparedness. This does not seem to go down well with the business community and may adversely impact the ease of doing business in India.

#### Functioning of the board as a digital office

Under the scheme the Act, the Data Protection Board (DPB) is envisaged as a digital office, raising concerns regarding access to justice. Rule 19 r/w Section 28 of the Act, provides that the Board shall function as a digital office “as far as practicable,” with the receipts of complaints and conduct of proceedings in an online/digital format. This design signals the efforts of the legislators to streamline the regulatory process and simultaneously reduce the administrative burden. However, given that the large segments of Indian population still lack access to reliable digital infrastructure, this model may potentially exclude such segments from availing the remedy or exercising their data protection rights. Although, personal data is owned by all persons across sections of the society, access to technology and reliable digital means remains exclusionary and inequitable. This asymmetry can further increase the digital divide and undermine the accessibility of the grievance redressal mechanism. Since, the Rules are at the Draught stage, there remains an opportunity to address this concern by incorporating an alternative for offline access to the grievance redressal mechanism. Such an arrangement would better align the Act’s implementation with the principles of procedural fairness and inclusiveness.

#### Research gap

A review of the existing scholarship reveals several empirical and comparative gaps in the study of the DPDP Act, 2023. There is an evident lack of comparative research on how different professional groups, such as lawyer, employees of corporations and bank employees engage with or interpret the provisions of the DPDP Act. Studies do not account for sectoral variation in understanding of the law, despite the fact that professional association determines one’s exposure to legal frameworks, compliance responsibilities, and provides an interpretative lens to engage with such legal compliance requirements. Secondly, empirical studies have not examined the institutional readiness of key sectors to comply with the Act. Thirdly, sparse data on the awareness level of rights available to individuals under the DPDP Act across professions necessitates a study of this kind. This is particularly relevant as awareness shapes expectations from the law and the Act emphasises on consent-based data governance model. Further, not sufficient academic attention has been paid to understand how professionals anticipate management of the operational trade-offs between privacy enforcement and corporation’s need for data processing.

Apart from the above, no empirical study has mapped the perspectives of stakeholders on the DPDP Act with respect to its effectiveness in addressing data privacy issues. Hence, this study seeks to conduct a context-sensitive, sector-wise analysis of the awareness, compliance and perceived effectiveness of the DPDP Act, 2023.

## Results

To examine the differences in privacy awareness across respondent groups, we hypothesised that there is no statistically significant difference in privacy awareness between employees of corporations and lawyers and laypersons (Supplementary Table S1).

An independent samples *t*-test was conducted to compare privacy awareness levels between two groups: (1) professionals (consisting of lawyers, employees of corporation and bank employees) and (2) laypersons. The results indicated a *statistically significant difference* in awareness levels, with employees of corporations and lawyers (*M* = 28.72, SD = 4.67) exhibiting higher privacy awareness than laypersons (*M* = 27.19, SD = 4.10), *t* (378) = 3.40, *p* = 0.001. The effect size (Cohen’s *d* = 0.34)^7^ suggests a small effect, indicating a *modest difference* in awareness between the two groups. Consequently, the hypothesis which posited no statistically significant difference in privacy awareness between professionals and laypersons, was rejected.

The findings suggest that professional exposure may contribute to higher awareness levels. However, given the small effect size, further investigation is needed to understand additional factors influencing awareness, such as education, training, or industry-specific exposure.

Building further on this insight, the analysis turns to the perceptions of the DPDP Act, 2023 to evaluate whether differences in awareness translate into divergent views on the awareness, effectiveness, implications of the law, amongst the two respondent groups.

### Awareness of rights under DPDP Act

With the objective of ascertaining whether awareness of rights under the DPDP Act, 2023 is independent of respondent’s professional association. We generated a cross tabulation (Supplementary Table S2) of the data using the IBM SPSS. The findings present a very compelling picture of how the awareness of the rights under the DPDP Act is at variance across different sub-groups of professionals. A strikingly high rate of respondents 55.4% demonstrated awareness of all three rights—rights to erasure, right to correction and the right to access. Although, this comprehensive awareness is not evenly distributed across segments of respondents. Employees of corporations, make up 59%, lawyers trail at 37.3% whilst bank employees were found lagging at merely 3.4%.

Further, analysed separately, the right to access emerged as comparatively most known, as 29.6% respondents acknowledged being aware of it. The employees of corporations aware of it comprised of (69.8%), followed by lawyers (23.8%), with bank employees staggering at 6.3%. The Right to Correction, though recognised by a smaller 7.5% of respondents, revealed an even starker divide—75.0% of those aware belonged to the corporate sector, whilst bank employees and lawyers contributed equally at 12.5% each. A similar pattern unfolded with the Right to Erase, with 62.5% of those aware being employees of corporations, 25.0% lawyers, and 12.5% bank employees.

These disparity or variance in responses suggest a larger picture, employees of corporations consistently showed higher awareness with the data protection rights, whilst bank employees remained largely unaware. The paradox lies in the fact that the lawyers who are trained in the regulatory framework do not exhibit highest level of awareness. This implies that exposure to data governance and handling on a daily basis, rather than legal expertise alone, plays a critical role in shaping one’s understanding of privacy rights.

The statistical analysis (see Supplementary Table S3) suggests that awareness of rights under the DPDP Act, 2023 is not at a significant variance. The *p*-value of 0.152 in chi-square tests exceeds the conventional threshold of 0.05. Consequently, we the hypothesis that awareness of rights is independent of one’s professional association could not be rejected. This implies that different professions—bank employees, employees of corporations, and lawyers, exhibit comparable levels of awareness of the rights envisaged under DPDP Act. The.

The absence of a statistically significant relationship suggests that factors other than professional affiliation, such as education, prior exposure to data privacy regulations, or access to relevant training, may play a more influential role in shaping awareness.

To ascertain whether awareness of rights under the DPDP Act was influenced if the corporations or organisations provided information about the Act, we conducted a chi-square test. We hypothesised that the awareness of rights under the DPDP Act, 2023 is independent of whether the corporation provided the respondent information on the DPDP Act, 2023.

### Awareness of rights and institutional communication

An analysis of the responses (see Supplementary Table S4) reveals an association between corporate communication and awareness of rights under the DPDP Act. Amongst those who reported receiving information about the Act from their employer, 63.7% indicated awareness of all the three rights. In contrast, 47.7% of the respondents who did not receive such communication indicated similar awareness. This suggests that institutional communicational can significantly enhance awareness.

Conversely, 63.5% of respondents who were aware only of the right to access, had not received any information about the Act from their corporation. This suggested that in absence of targeted communication, employees are aware only of the most basic or most discussed right. With respect to the right to correction, 62.5% of the respondents who were aware of it had received corporate communication about the Act. This implies that awareness about technical rights is shaped by formal communication. However, in relation to awareness of right to erasure, the trend depicts a different picture. A substantial 75% respondents who were aware of this right had not received information from their organisation on DPDP Act. This reflects general uncertainty or limited organisational emphasis on this right. Viewed together, the findings demonstrate that institutional engagement or corporate communication can shape the awareness of data protection rights, particularly with respect to more nuanced or technical rights.

Since, the value of *p* generated by Chi-square test (Supplementary Table S5) is 0.015 (below 0.05), the null hypothesis of independence is rejected. This implies that there is a statistically significant association between information dissemination by the corporation and the awareness of rights amongst employees.

### Perceived challenges in implementing the DPDP Act, 2023

The SPSS software was used to generate a crosstabulation (Supplementary Table S6) between implementation challenges and corporation’s efforts to comply with the DPDP Act, 2023. The crosstab revealed many key patterns in organisational preparedness. Compliance monitoring and reporting emerged on top most challenging (36.4%) with more than half (54.5%) respondents agreeing or strongly agreeing that corporation is undertaking compliance measures. This indicates that the organisations actively engaged in compliance may be more conversant with the regulatory demands and thus perceive compliance monitoring as most challenging. Similarly, technical challenges (15.5%) and financial costs (8.5%) were also significant concerns, particularly amongst respondents who perceived their corporations as less engaged in compliance.

Interestingly, 30.2% respondents who cited technical challenges as most challenging, strongly disagreed that their corporation is taking steps to comply, indicating a potential link between weak or rather lesser compliance engagement and technical difficulty. Similarly, those who identified financial costs as the primary barrier, reported lesser agreement with corporate compliance efforts. This suggests that financial constraints may hinder legal compliances.

Perceptions pertaining to the change in business processes (16.9%) and staff training appeared to be most evenly distributed. A sizable population gave neutral response on it. This reflects early-stage engagement with this new law, where corporations are yet to assess operational impact and encounter new challenges.

Overall, the response crosstabulation indicates that awareness of specific challenges to compliance varies as per corporation’s efforts to comply with the law.

The *p* value of 0.003 generated by the Chi-square test (Supplementary Table S7) revealed significant association between corporation’s compliance efforts and the perceived challenges in implementing the DPDP Act. This suggests that the perception of compliance efforts is meaningfully linked to how individuals assess specific implementation barriers.

### Perceptions of ease of compliance of DPDP Act across respondent groups

We tried to ascertain whether the perceptions of the DPDP Act as an easy to comply law depends largely on one’s professional association or not (Supplementary Table S8). It was found that 81.4% of employees of corporations strongly disagreed that the law was easy to comply, signalling a broad perception of regulatory difficulty amongst this group. Lawyers, however, held a balanced view with 41.8% were neutral, 39.4% agreed that law was easy to comply, indicating a broad perception of regulatory difficulty amongst corporate professionals. Bank employees gave mixed responses with 50% disagreeing and 16.7% agreeing, and none strongly agreed to the proposition that DPDP Act is an easy to comply law. Notably, respondents who strongly agreed were employees of corporations, though in a miniscule minority of 1.4% of the total sample. The *p* value of 0.023 which chi-square test (Supplementary Table S9) yielded, depicts a significant association between profession and perception of DPDP Act’s ease of compliance. This, led to the rejection of the null hypothesis of independence.

### Perceptions of effectiveness in balancing business and privacy

The findings (see Supplementary Table S10) reveal that the three professional groups vary in their perception of DPDP Act’s effectiveness in balancing data protection with the need of businesses and corporations to process data. In the category of bank employees, 66.6% either strongly disagreed or disagreed and only 16.6% expressed their agreement, signalling scepticism. Similarly, employees of corporations exhibited highest disagreement, outlining a general perception that the Act is likely to impose operational burdens on business processes. In contrast, lawyers had a balanced view with 41.5 disagreeing 21.5% agreeing and 36.9% remained neutral to the Act’s effectiveness in this regard—suggesting a rather cautious approach towards regulatory framework.

A *p*-value of 0.074 was yielded by the Chi-square test of independence (Supplementary Table S11). As this exceeds the conventional threshold of 0.05, we failed to reject the null hypothesis. This implies that the there is no statistically significant relationship between professional association and the perception of the Act’s effectiveness in balancing the needs of data protection and business.

### Ease of doing business

Whether one’s perception that DPDP Act, 2023 facilitates ease of doing business is independent of one’s professional association was examined. 66.7% of the bank employees disagreed (25.0 strongly disagreed and 41.7% disagreed), 54.5% of employees w corporations (22.1% strongly disagreed and 32.4% disagreed) whilst 36.8% were neutral. The lawyers also mirrored the same trend with 52.3% disagreeing (16.9% strongly disagreed and 35.4% agreed) and 38.5% were neutral—the highest amongst the three set of respondents. Notably, agreement levels were comparatively low across respondents, pointing that the Act is not perceived as business-friendly. The Chi-square test (Supplementary Table S13) yielded a *p*-value of 0.082, which above the threshold. Consequently, we failed to reject the null hypothesis of no statistically significant association between professional background and the perception of the Act as fostering ease of doing business.

### Fostering innovation

On the proposition that DPDP Act fosters innovation, the bank employees exhibited highest scepticism as 66.7% disagreed (16.7% strongly disagreed and 50.0% disagreed), whilst 59.6% of the employees of corporations disagreed (20.6% strongly disagreed and 39.0% disagreed) and only 13.2% expressed agreement (8.8% strongly agreed and 4.4% agreed). In the category of lawyers, disagreement or scepticism was comparatively lower as 46.2% of them disagreed (15.4% strongly disagreed and 30.8% disagreed), however, neutral responses were highest in this group at 43.1% (See Supplementary Table S14).

Further, chi-square test of independence (Supplementary Table S15) yielded a *p*-value of 0.319 suggesting that there is no statistically significant association between the respondent’s perception about the Act as fostering innovation and their professional association.

### Grievance redressal mechanism

The belief that the grievance redressal mechanism is independent of professional association was analysed to see if it varies across the respondent groups (Supplementary Table S15). The bank employees showed highest disagreement—66.7% disagreed (16.7% strongly disagreed and 50.0% disagreed), followed by employees of corporations 58.8% disagreed (25.0% strongly disagreed and 33.8% disagreed), lawyers, on the contrary were balanced in their perception. 35.4% disagreed (12.3% strongly disagreed and 23.1% disagreed) and 50.8% were neutral in their stance whilst only 10.8% strongly agreed and 2.2% agreed. The high level of neutral responses indicates their perception is likely to be contingent upon practical application of the law and the future judicial pronouncements.

Kendall’s tau-b test (see Supplementary Table S16) was conducted to determine the direction of association amongst variables. It yielded a value of *p* = 0.004 which is statistically significant. This suggests a weak yet meaningful ordinal association between the professional background and the perception about the effectiveness of the grievance redressal mechanism.

### Effectiveness of the law in safeguarding data privacy

On analysing the responses to the statement that ‘DPDP Act will be effective in addressing my concerns regarding data privacy’, difference of opinion across respondent groups was observed (Supplementary Table S17). Bank employees exhibited the lowest level of confidence in the effectiveness of the Act with 66.7% (41.7% strongly disagreed and 25.0% disagreed), only 16.7% expressed agreement and the same number were neutral, whilst none expressed strong agreement. This indicates that the financial sector professionals perceive regulatory gaps or challenges in implementation of this Act. The employees of corporations, on the other hand, were divided in their stand, 60.9% disagreed (31.6% strongly disagreed and 29.4% disagreed), and 30.1% remained neutral, whilst 8.8% agreed (5.9% strongly agreed and 2.9% agreed). Amongst the group of lawyers, 38.5% showed neutrality of response, 44.6 disagreed (16.9% strongly disagreed and 27.7% disagreed) and 16.9% agreed—suggesting a relatively higher level of confidence in the Act.

Chi-square test of independence (Supplementary Table S18) was conducted to identify the significance of association. It gave a *p*-value of 0.067 which is slightly above the conventional threshold. This implies that there is a marginal association and not statistically significant association between the professional association and perception about Act’s effectiveness in addressing data privacy concerns. Hence, the null hypothesis is accepted.

### Repeal of distinction

An analysis of the data on perception of professionals on ‘repeal of Section 43A of the IT Act and elimination of the distinction between sensitive personal data and personal data under the DPDP Act, 2023 as justified’, is independent of the respondents’ professional background (bank employees, employees of corporations, and lawyers) (see Supplementary Table S19).

The findings indicate that most professionals strongly disagree with the justification for repealing Section 43A of the IT Act and eliminating the distinction between sensitive personal data and personal data under the DPDP Act, 2023 is not independent of the professional association. Different professionals perceive this differently. Lawyers rejected this change and demonstrated significant opposition with 59.3% disagreeing (22.8% strongly disagreeing, 36.5% disagreeing). Only 30.8% (15.4% strongly agree, 15.4% agree) support it, whilst a small proportion remains neutral. The strong disagreement amongst legal professionals suggests that they view the removal of this distinction as a weakening of data protection safeguards rather than a move towards regulatory clarity.

Employees of corporations also predominantly opposed the change, with 49.3% (22.8% strongly disagreeing, 26.5% disagreeing). Only 21.3% (6.6% strongly agree, 14.7% agree) believe the change is justified, whilst 29.4% remain neutral. This widespread opposition reflects concerns within the corporate sector regarding data security risks, compliance challenges, and potential liabilities arising from the elimination of differentiated data protection standards.

Bank employees exhibited the strongest opposition, with 75% (33.3% strongly disagree, 41.7% disagree) outrightly rejecting the removal of the distinction. Notably, no bank employees agreed with the change, and 25% remained neutral. The unanimous disapproval within this group suggests that financial professionals view the distinction between personal and sensitive personal data as critical for regulatory compliance, fraud prevention, and consumer protection.

As strong disagreement is witnesses across the groups, this implies that the DPDP Act’s intent to repeal of Section 43A of the IT Act and the removal of the distinction between personal and sensitive personal data, are perceived as unjustified by a major section of respondents. This signals the resistance across sectors, flagging serious concerns of data privacy, security and the adequacy of the protective framework.

The chi-square test results indicate a *p*-value of 0.127, which exceeds the conventional significance threshold of 0.05. This suggests that the perception of whether the repeal of Section 43A of the IT Act and the elimination of the distinction between sensitive personal data and personal data under the DPDP Act, 2023, is justified is independent of the respondents’ professional background. In other words, there is no statistically significant association between a respondent’s profession (lawyer, corporate employee, or bank employee) and their stance on this issue.

## Discussion

The empirical findings of the study have to be located within the constitutional and theoretical landscape of privacy in India—as shaped by the landmark *Puttaswamy* (9j.) judgement and the articulation of informational privacy as a fundamental right. The principles of autonomy, self-determination and state accountability outlined in the said judgement have become normative benchmarks on the anvil of which, the legislative actions like the DPDP Act, 2023 have to be evaluated. Similarly, theories of privacy, especially those that perceive privacy as something related to human dignity (similar to the court’s opinion in *Puttaswamy* judgement) or emphasise on the appropriateness of flow of information within specific contexts (as in Nissenbaum’s contextual integrity) are crucial for understanding how privacy protections translate or function in practise. These frameworks explain the experiences of professionals captured through questionnaires for this study, showing that the legal rights alone are insufficient unless supported by a conducive institutional ecosystem that empowers individuals with the ability to exercise those rights.

The empirical patterns observed highlight an asymmetry between the legal recognition of the privacy right and the actual experiences of professionals across sectors. Whilst the DPDP Act, 2023 intends to fulfil the constitutional mandate of protecting personal data, the findings suggest that Act’s perceived efficacy is uneven, specifically in terms of awareness of rights, perceived ease of its compliance and corporations’ trust in the enforcement mechanisms envisaged. The data suggests that only 55.4% of professionals demonstrated awareness of all the three rights, erasure, access and correction. The employees of corporations were found to be significantly more aware than the bank employees or lawyers. Despite such variation, the chi-square test results established that professional background is not the sole determinant of awareness (*p* = 0.152). Nonetheless, the provision of information about DPDP Act supplied by the organisations/corporations was strongly associated with high awareness levels (*p* = 0.015). In a similar pattern, employees who indicated that their corporations are taking efforts to comply with the law were more likely to identify nuanced implementation challenges. This indicates a correlation between institutional preparedness and legal consciousness (*p* = 0.003).

These patterns suggest that the legal recognition or guarantee of rights, does not automatically translate into capabilities for exercising such rights. This mirrors the Nobel laurate Amartya Sen’s capabilities approach, which postulates that the effectiveness of a particular right depends on whether individuals possess the requisite support—institutional, social and informational, to realise that right. Similarly, as Martha Nussbaum asserts that one’s ability to control their environment including the control over information is critical to human dignity.^8^

The low levels of awareness amongst legal professionals and bank employees underscore a capability gap that cannot be addressed by legal formalism or recognition alone. Further, data affirms Helen Nissenbaum’s contextual integrity theory, which does not articulate privacy as an ‘abstract control over data’ rather ‘as the appropriateness of information flows’ in given contexts. The employees of corporations working with compliance driven organisational setups are more exposed to the norms of privacy and the data handling practises. Conversely, bank employees and lawyers, who although are part of regulated sectors, appear to operate in environments where data protection law and rules are comparatively less disseminated. Thus, awareness about the law is not merely contingent upon one’s professional expertise rather upon whether information about the laws is communicated to the professionals and they are routinely complied in operations.

The findings also raise questions pertaining to the two constitutional standards identified in the *Puttaswamy* judgement—proportionality and substantive equality. The corporate employees seem to have felt disproportionate burden with 81.4% strongly disagreeing that the DPDP law is an easy to comply law. This is juxtaposed with the limited institutional information and support provided in other sector, suggesting a very skewed landscape of implementation/compliance.

There is no statistically significant association between professional background and perceptions regarding innovation, ease of doing business or on the Act’s balancing with privacy. This indicates lack of consensus or clarity on broader economic implications of the law. It can also be inferred from the data that whilst legal theory considers privacy as essential for innovation and trust in the operation of digital economies, empirical data shows a different picture that such narratives have not permeated the understanding of professionals.

Finally, the weak but statistically significant association (*p* = 0.004) between professional background and perceptions of grievance redressal effectiveness reflects limited confidence in the enforceability of the DPDP Act’s protective mechanisms. Given the centrality of access to remedies in the architecture of fundamental rights, this perception gap challenges the legitimacy and normative completeness of the law’s operational design.

*In toto*, the empirical findings indicate that DPDP Act’s promise of giving individuals control over their personal data remains unevenly realised. If the right to privacy has to be more than a mere theoretical entitlement, the operationalisation of this right must account for institutional, contextual and informational asymmetries. The law must therefore be supported by capacity building measures and differentiated communication strategies which are in tandem with professional contexts where processing of data occurs.

By anchoring implementation in the lived experiences of the professionals who deal with the data, the Act can enhance its effectiveness. This would require conscious efforts towards bridging the gap between formal rights and real freedom—a metamorphosis necessary for realising the constitutional guarantee.

## Conclusion

The strongest protection for an individual’s privacy lies in constitutional guarantees. India affords such protection by recognising the right to privacy as a fundamental right and the informational privacy as the dimension of former. The data protection law enacted post-recognition of privacy as fundamental right, i.e., the DPDP Act, claims to extend a catena of rights to the individuals, including the control over personal information, right to access, correction, erasure of data, amongst other things. However, the effectiveness of these rights, is contingent upon institutional readiness, accessibility and awareness amongst the users as well as the professionals who handle personal data. Findings of this study shows considerable variation in the awareness levels of lawyers, bank employees and employees of corporations. Where some respondents were familiar with these rights, a large section expressed uncertainty about their enforceability and redressal mechanism in case of infringement. The wide gap between having the rights and their practical understanding raises serious questions about implementation, outreach, and sector-specific compliance under the DPDP Act.

This study positions itself as anticipatory, given the nascent stage of the DPDP Act. Since the Act is yet to be fully operationalised, an assessment of its normative design and projected implications is crucial for informing law and policy making shaping legal debates and preparing relevant stakeholders for compliance. It does not aim to predict any immediate practical effects, rather it seeks to evaluate the normative aspirations of law, its institutional requirements and its possible trajectory within India’s evolving data governance ecosystem. Just as the pre-enforcement scholarship contributed in effective enforcement of the Right to Information Act, 2005, a similar scholarly engagement with the DPDP Act, will catalyse smoother enforcement, guide institutional preparedness and increase public awareness.

In this backdrop, the study concludes that although the DPDP law lays a foundational legal architecture for data protection in India, its perceived legitimacy and effectiveness remain uneven. The difference of opinions reflects not only sectoral differences but also the exposure to the regulatory framework. They reinforce the significance of aligning the law with the practical realities. As this nascent law and the Indian data governance framework continue to evolve more empirical studies would be required to ascertain the impact and effectiveness of the law in protecting data.

## Data Availability

The raw data supporting the conclusions of this article will be made available by the authors, without undue reservation.

## Footnotes

1 The Supreme Court recognised privacy as a fundamental right under arts 14, 19 and 21 of the Constitution, and laid down a three-part test for any infringement: legality, legitimate state aim, and proportionality. The Court further called for a structured data protection regime grounded in the principles of informational self-determination, autonomy, and dignity.

2 The Information Technology Act, 2000 did not define *personal* data. However, Rule 2(1)(i) of the SPDI Rules defined personal *information.* Although, the IT Act defines ‘information’ and ‘data’ separately, there are synonymously used in the Act.

3 Section 7. A Data Fiduciary may process personal data of a Data Principal for any of following uses, namely: (a) For the specified purpose for which the Data Principal has voluntarily provided her personal data to the Data Fiduciary, and in respect of which she has not indicated to the Data Fiduciary that she does not consent to the use of her personal data.

4 Section 7(b) for the State and any of its instrumentalities to provide or issue to the Data Principal such subsidy, benefit, service, certificate, licence or permit as may be prescribed, where: (i) She has previously consented to the processing of her personal data by the State or any of its instrumentalities for any subsidy, benefit, service, certificate, licence or permit; or (ii) Such personal data is available in digital form in, or in non-digital form and digitised subsequently from, any database, register, book or other document which is maintained by the State or any of its instrumentalities and is notified by the Central Government, subject to standards followed for processing being in accordance with the policy issued by the Central Government or any law for the time being in force for governance of personal data.

5 Rule 22. Calling for information from Data Fiduciary or intermediary. (1) The Central Government may, for such purposes of the Act as are specified in Seventh Schedule, acting through the corresponding authorised person specified in the said Schedule, require any Data Fiduciary or intermediary to furnish such information as may be called for, specify the time period within which the same shall be furnished and, where disclosure in this regard is likely to prejudicially affect the sovereignty and integrity of India or security of the State, require the Data Fiduciary or intermediary to not disclose the same except with the previous permission in writing of the authorised person. (2) Provision of information called for under this rule shall be by way of fulfilment of obligation under section 36 of the Act.

6 Section 12. (1) A Data Principal shall have the right to correction, completion, updating and erasure of her personal data for the processing of which she has previously given consent, including consent as referred to in clause (a) of section 7, in accordance.

7 Cohen’s *d* is a measure of effect size that quantifies the magnitude of the difference between two groups in terms of standard deviations. Cohen’s conventional benchmarks:

- Small effect: *d* = 0.2.
- Medium effect: *d* = 0.5.
- Large effect: *d* = 0.8.

8 The capability approach developed by Nobel laureate Amartya Sen and academic Martha Nussbaum, offers a very compelling theoretical framework for evaluating the data protection legislations. With respect to the DPDP Act, 2023, the capability approach reveals that mere provision of rights is inadequate. It is for the State and corporations to facilitate the exercise of rights by individuals through raising awareness, procedural support, accessible enforcement mechanisms. This theoretical framework is important for understanding the distinction and disparity between the recognition of the right and its exercise and awareness.
