# Supplementary material for: Stakeholder perceptions of India’s Digital Personal Data Protection Act of 2023: an empirical study across legal, banking, and corporate sectors
Source: Front Sociol. 2026 May 29;11:1753383. doi: 10.3389/fsoc.2026.1753383 (PMC13259712; doi:10.3389/fsoc.2026.1753383)
Supplement: Supplementary file 1 [file Supplementary_file_1.docx]

**SUPPLEMENTARY MATERIAL**

**Table 02: Cross Tabulation**

| **Q22 (Rights)** | **Bank Employees** | **Employee of Corporations** | **Lawyers** | **Total** |
| --- | --- | --- | --- | --- |
| **Right to Erase** | | | | |
| % within Q22 | 12.5% | 62.5% | 25.0% | 100.0% |
| % within Q8^[[1]](#footnote-1)^ | 16.7% | 7.4% | 6.2% | 7.5% |
| % of Total | 0.9% | 4.7% | 1.9% | 7.5% |
| **Right to Correction** | | | | |
| % within Q22 | 12.5% | 75.0% | 12.5% | 100.0% |
| % within Q8 | 16.7% | 8.8% | 3.1% | 7.5% |
| % of Total | 0.9% | 5.6% | 0.9% | 7.5% |
| **Right to Access** | | | | |
| % within Q22^[[2]](#footnote-2)^ | 6.3% | 69.8% | 23.8% | 100.0% |
| % within Q8 | 33.3% | 32.4% | 23.1% | 29.6% |
| % of Total | 1.9% | 20.7% | 7.0% | 29.6% |
| **All of the Above** | | | | |
| % within Q22 | 3.4% | 59.3% | 37.3% | 100.0% |
| % within Q8 | 33.3% | 51.5% | 67.7% | 55.4% |
| % of Total | 1.9% | 32.9% | 20.7% | 55.4% |
| **Total Count** | | | | |
| % within Q22 | 5.6% | 63.8% | 30.5% | 100.0% |
| % within Q8 | 100.0% | 100.0% | 100.0% | 100.0% |
| % of Total | 5.6% | 63.8% | 30.5% | 100.0% |

**Table 03: Result of Chi-square (𝜒²) Tests**

| **Test** | **Value** | **df** | **Asymptotic Significance (2-sided)** |
| --- | --- | --- | --- |
| Pearson Chi-square (𝜒²) | 9.402 | 6 | 0.152 |

## Table 04: Cross Tabulation

|  | | | Q35 | | Total |
| --- | --- | --- | --- | --- | --- |
|  |  |  | No | Yes |  |
| Q22 | All of the above | % within Q22 | 44.9% | 55.1% | 100.0% |
|  |  | % within Q35^[[3]](#footnote-3)^ | 47.7% | 63.7% | 55.4% |
|  |  | % of Total | 24.9% | 30.5% | 55.4% |
|  | Right to access | % within Q22 | 63.5% | 36.5% | 100.0% |
|  |  | % within Q35 | 36.0% | 22.5% | 29.6% |
|  |  | % of Total | 18.8% | 10.8% | 29.6% |
|  | Right to correction | % within Q22^[[4]](#footnote-4)^ | 37.5% | 62.5% | 100.0% |
|  |  | % within Q35 | 5.4% | 9.8% | 7.5% |
|  |  | % of Total | 2.8% | 4.7% | 7.5% |
|  | Right to erase | % within Q22 | 75.0% | 25.0% | 100.0% |
|  |  | % within Q35 | 10.8% | 3.9% | 7.5% |
|  |  | % of Total | 5.6% | 1.9% | 7.5% |
| Total | | % within Q22 | 52.1% | 47.9% | 100.0% |
|  |  | % within Q35 | 100.0% | 100.0% | 100.0% |
|  |  | % of Total | 52.1% | 47.9% | 100.0% |

**Table 05**: **Result of Chi-square (𝜒²) Tests**

| **Test** | **Value** | **df** | **Asymp. Sig. (2-sided)** |
| --- | --- | --- | --- |
| Pearson Chi-square (𝜒²) | 10.446 | 3 | 0.015 |

**Table 06: Cross Tabulation**

|  | | | Q36 | | | | | |
| --- | --- | --- | --- | --- | --- | --- | --- | --- |
|  |  |  | Strongly Disagree | Disagree | Neutral | Agree | Strongly agree | Total |
| Q31 | Changes to existing business processes | % within Q31 | 16.7% | 47.2% | 25.0% | 5.6% | 5.6% | 100.0% |
|  |  | % within Q36 | 11.3% | 28.8% | 13.8% | 8.0% | 18.2% | 16.9% |
|  |  | % of Total | 2.8% | 8.0% | 4.2% | 0.9% | 0.9% | 16.9% |
|  | Compliance monitoring and reporting | % within Q31^[[5]](#footnote-5)^ | 18.2% | 19.5% | 36.4% | 18.2% | 7.8% | 100.0% |
|  |  | % within Q36^[[6]](#footnote-6)^ | 26.4% | 25.4% | 43.1% | 56.0% | 54.5% | 36.2% |
|  |  | % of Total | 6.6% | 7.0% | 13.1% | 6.6% | 2.8% | 36.2% |
|  | Financial costs | % within Q31 | 44.4% | 33.3% | 22.2% | 0.0% | 0.0% | 100.0% |
|  |  | % within Q36 | 15.1% | 10.2% | 6.2% | 0.0% | 0.0% | 8.5% |
|  |  | % of Total | 3.8% | 2.8% | 1.9% | 0.0% | 0.0% | 8.5% |
|  | Staff training and awareness | % within Q31 | 18.4% | 26.5% | 36.7% | 16.3% | 2.0% | 100.0% |
|  |  | % within Q36 | 17.0% | 22.0% | 27.7% | 32.0% | 9.1% | 23.0% |
|  |  | % of Total | 4.2% | 6.1% | 8.5% | 3.8% | 0.5% | 23.0% |
|  | Technical challenges | % within Q31 | 48.5% | 24.2% | 18.2% | 3.0% | 6.1% | 100.0% |
|  |  | % within Q36 | 30.2% | 13.6% | 9.2% | 4.0% | 18.2% | 15.5% |
|  |  | % of Total | 7.5% | 3.8% | 2.8% | 0.5% | 0.9% | 15.5% |
| Total | % within Q31 | | 24.9% | 27.7% | 30.5% | 11.7% | 5.2% | 100.0% |
|  | % within Q36 | | 100.0% | 100.0% | 100.0% | 100.0% | 100.0% | 100.0% |
|  | % of Total | | 24.9% | 27.7% | 30.5% | 11.7% | 5.2% | 100.0% |

**Table 07: Result of Chi-square (𝜒²) Tests**

| Test | Value | df | Asymp. Sig. (2-sided) |
| --- | --- | --- | --- |
| Pearson Chi-square (𝜒²) | 36.423 | 16 | 0.003 |

## Table 08: Cross Tabulation

|  | | | Q8 | | | Total |
| --- | --- | --- | --- | --- | --- | --- |
|  |  |  | Bank Employees | Employees of corporation | Lawyers |  |
| Q34 | Strongly Disagree | % within Q34 | 2.3% | 81.4% | 16.3% | 100.0% |
|  |  | % within Q8 | 8.3% | 25.7% | 10.8% | 20.2% |
|  |  | % of Total | 0.5% | 16.4% | 3.3% | 20.2% |
|  | Disagree | % within Q34 | 10.9% | 67.3% | 21.8% | 100.0% |
|  |  | % within Q8 | 50.0% | 27.2% | 18.5% | 25.8% |
|  |  | % of Total | 2.8% | 17.4% | 5.6% | 25.8% |
|  | Neutral | % within Q34 | 3.8% | 54.4% | 41.8% | 100.0% |
|  |  | % within Q8 | 25.0% | 31.6% | 50.8% | 37.1% |
|  |  | % of Total | 1.4% | 20.2% | 15.5% | 37.1% |
|  | Agree | % within Q34 | 6.1% | 54.5% | 39.4% | 100.0% |
|  |  | % within Q8 | 16.7% | 13.2% | 20.0% | 15.5% |
|  |  | % of Total | 0.9% | 8.5% | 6.1% | 15.5% |
|  | Strongly Agree | % within Q34 | 0.0% | 100.0% | 0.0% | 100.0% |
|  |  | % within Q8 | 0.0% | 2.2% | 0.0% | 1.4% |
|  |  | % of Total | 0.0% | 1.4% | 0.0% | 1.4% |
| Total | | % within Q34^[[7]](#footnote-7)^ | 5.6% | 63.8% | 30.5% | 100.0% |
|  |  | % within Q8 | 100.0% | 100.0% | 100.0% | 100.0% |
|  |  | % of Total | 5.6% | 63.8% | 30.5% | 100.0% |

**Table 09: Result of Chi-square (𝜒²) Tests**

| **Test** | **Value** | **df** | **Asymp. Sig. (2-sided)** |
| --- | --- | --- | --- |
| Pearson Chi-square (𝜒²) | 17.805 | 8 | 0.023 |

## Table 10: Cross Tabulation

|  | | | Q24 | | | |  |  |
| --- | --- | --- | --- | --- | --- | --- | --- | --- |
|  |  |  | Strongly Disagree | Disagree | Neutral | Agree | Strongly Agree | Total |
| Q8 | Bank Employees | % within Q8 | 33.3% | 33.3% | 16.7% | 8.3% | 8.3% | 100.0% |
|  |  | % within Q24^[[8]](#footnote-8)^ | 9.8% | 4.9% | 3.4% | 4.5% | 11.1% | 5.6% |
|  |  | % of Total | 1.9% | 1.9% | 0.9% | 0.5% | 0.5% | 5.6% |
|  | Employees of corporations | % within Q8 | 22.8% | 41.9% | 24.3% | 7.4% | 3.7% | 100.0% |
|  |  | % within Q24 | 75.6% | 69.5% | 55.9% | 45.5% | 55.6% | 63.8% |
|  |  | % of Total | 14.6% | 26.8% | 15.5% | 4.7% | 2.3% | 63.8% |
|  | Lawyers | % within Q8 | 9.2% | 32.3% | 36.9% | 16.9% | 4.6% | 100.0% |
|  |  | % within Q24 | 14.6% | 25.6% | 40.7% | 50.0% | 33.3% | 30.5% |
|  |  | % of Total | 2.8% | 9.9% | 11.3% | 5.2% | 1.4% | 30.5% |
| Total | | % within Q8 | 19.2% | 38.5% | 27.7% | 10.3% | 4.2% | 100.0% |
|  |  | % within Q24 | 100.0% | 100.0% | 100.0% | 100.0% | 100.0% | 100.0% |
|  |  | % of Total | 19.2% | 38.5% | 27.7% | 10.3% | 4.2% | 100.0% |

**Table 11: Result of Chi-square (𝜒²) Tests**

| **Test** | **Value** | **df** | **Asymp. Sig. (2-sided)** |
| --- | --- | --- | --- |
| Pearson Chi-square (𝜒²) | 14.307 | 8 | 0.074 |

# Table 12: Cross Tabulation

|  | | | Q26 | | | |  |  |
| --- | --- | --- | --- | --- | --- | --- | --- | --- |
|  |  |  | Strongly Disagree | Disagree | Neutral | Agree | Strongly Agree | Total |
| Q8 | Bank Employees | % within Q8 | 25.0% | 41.7% | 8.3% | 25.0% | 0.0% | 100.0% |
|  |  | % within Q26^[[9]](#footnote-9)^ | 6.8% | 6.9% | 1.3% | 18.8% | 0.0% | 5.6% |
|  |  | % of Total | 1.4% | 2.3% | 0.5% | 1.4% | 0.0% | 5.6% |
|  | Employees of corporations | % within Q8 | 22.1% | 32.4% | 36.8% | 5.9% | 2.9% | 100.0% |
|  |  | % within Q26 | 68.2% | 61.1% | 65.8% | 50.0% | 80.0% | 63.8% |
|  |  | % of Total | 14.1% | 20.7% | 23.5% | 3.8% | 1.9% | 63.8% |
|  | Lawyers | % within Q8 | 16.9% | 35.4% | 38.5% | 7.7% | 1.5% | 100.0% |
|  |  | % within Q26 | 25.0% | 31.9% | 32.9% | 31.2% | 20.0% | 30.5% |
|  |  | % of Total | 5.2% | 10.8% | 11.7% | 2.3% | 0.5% | 30.5% |
| Total | | % within Q8 | 20.7% | 33.8% | 35.7% | 7.5% | 2.3% | 100.0% |
|  |  | % within Q26 | 100.0% | 100.0% | 100.0% | 100.0% | 100.0% | 100.0% |
|  |  | % of Total | 20.7% | 33.8% | 35.7% | 7.5% | 2.3% | 100.0% |

**Table 13:** **Result of Chi-square (𝜒²) Tests**

| **Test** | **Value** | **df** | **Asymp. Sig. (2-sided)** |
| --- | --- | --- | --- |
| Pearson Chi-square (𝜒²) | 9.767^a^ | 8 | .282 |

# Table 14: Cross Tabulation

|  | | | Q27 | | | |  |  |
| --- | --- | --- | --- | --- | --- | --- | --- | --- |
|  |  |  | Strongly Disagree | Disagree | Neutral | Agree | Strongly Agree | Total |
| Q8 | Bank Employees | % within Q8 | 16.7% | 50.0% | 25.0% | 8.3% | 0.0% | 100.0% |
|  |  | % within Q27^[[10]](#footnote-10)^ | 5.0% | 7.6% | 4.4% | 5.0% | 0.0% | 5.6% |
|  |  | % of Total | 0.9% | 2.8% | 1.4% | 0.5% | 0.0% | 5.6% |
|  | Employees of corporations | % within Q8 | 20.6% | 39.0% | 27.2% | 8.8% | 4.4% | 100.0% |
|  |  | % within Q27 | 70.0% | 67.1% | 54.4% | 60.0% | 100.0% | 63.8% |
|  |  | % of Total | 13.1% | 24.9% | 17.4% | 5.6% | 2.8% | 63.8% |
|  | Lawyers | % within Q8 | 15.4% | 30.8% | 43.1% | 10.8% | 0.0% | 100.0% |
|  |  | % within Q27 | 25.0% | 25.3% | 41.2% | 35.0% | 0.0% | 30.5% |
|  |  | % of Total | 4.7% | 9.4% | 13.1% | 3.3% | 0.0% | 30.5% |
| Total | | % within Q8 | 18.8% | 37.1% | 31.9% | 9.4% | 2.8% | 100.0% |
|  |  | % within Q27 | 100.0% | 100.0% | 100.0% | 100.0% | 100.0% | 100.0% |
|  |  | % of Total | 18.8% | 37.1% | 31.9% | 9.4% | 2.8% | 100.0% |

**Table 15: Result of Chi-square (𝜒²) Tests**

| **Test** | **Value** | **df** | **Asymp. Sig. (2-sided)** |
| --- | --- | --- | --- |
| Pearson Chi-square (𝜒²) | 9.284 | 8 | 0.319 |

## Table 15: Cross Tabulation

|  | | | Q33^[[11]](#footnote-11)^ | | | |  |  |
| --- | --- | --- | --- | --- | --- | --- | --- | --- |
|  |  |  | Strongly Disagree | Disagree | Neutral | Agree | Strongly Agree | Total |
| Q8 | Bank Employees | % within Q8 | 16.7% | 50.0% | 25.0% | 8.3% | 0.0% | 100.0% |
|  |  | % within Q33 | 4.5% | 9.0% | 4.0% | 4.5% | 0.0% | 5.6% |
|  |  | % of Total | 0.9% | 2.8% | 1.4% | 0.5% | 0.0% | 5.6% |
|  | Employees of corporations | % within Q8 | 25.0% | 33.8% | 28.7% | 10.3% | 2.2% | 100.0% |
|  |  | % within Q33 | 77.3% | 68.7% | 52.0% | 63.6% | 60.0% | 63.8% |
|  |  | % of Total | 16.0% | 21.6% | 18.3% | 6.6% | 1.4% | 63.8% |
|  | Lawyers | % within Q8 | 12.3% | 23.1% | 50.8% | 10.8% | 3.1% | 100.0% |
|  |  | % within Q33 | 18.2% | 22.4% | 44.0% | 31.8% | 40.0% | 30.5% |
|  |  | % of Total | 3.8% | 7.0% | 15.5% | 3.3% | 0.9% | 30.5% |
| Total | | % within Q8 | 20.7% | 31.5% | 35.2% | 10.3% | 2.3% | 100.0% |
|  |  | % within Q33 | 100.0% | 100.0% | 100.0% | 100.0% | 100.0% | 100.0% |
|  |  | % of Total | 20.7% | 31.5% | 35.2% | 10.3% | 2.3% | 100.0% |

**Table 16: Results of the Kendall's tau-b**

| **Test** | | **Value** | **Asymp. Std. Error** | **Approx. T** | **Approx. Sig.** |
| --- | --- | --- | --- | --- | --- |
| Ordinal by Ordinal | Kendall's tau-b | 0.167 | 0.057 | 2.915 | 0.004 |

# Table 17: Cross Tabulation

|  | | | Q21 | | | | | |
| --- | --- | --- | --- | --- | --- | --- | --- | --- |
|  |  |  | Strongly Disagree | Disagree | Neutral | Agree | Strongly Agree | Total |
| Q8 | Bank Employees | % within Q8 | 41.7% | 25.0% | 16.7% | 16.7% | 0.0% | 100.0% |
|  |  | % within Q21^[[12]](#footnote-12)^ | 8.5% | 4.9% | 2.9% | 9.5% | 0.0% | 5.6% |
|  |  | % of Total | 2.3% | 1.4% | 0.9% | 0.9% | 0.0% | 5.6% |
|  | Employees of corporations | % within Q8 | 31.6% | 29.4% | 30.1% | 5.9% | 2.9% | 100.0% |
|  |  | % within Q21 | 72.9% | 65.6% | 60.3% | 38.1% | 100.0% | 63.8% |
|  |  | % of Total | 20.2% | 18.8% | 19.2% | 3.8% | 1.9% | 63.8% |
|  | Lawyers | % within Q8 | 16.9% | 27.7% | 38.5% | 16.9% | 0.0% | 100.0% |
|  |  | % within Q21 | 18.6% | 29.5% | 36.8% | 52.4% | 0.0% | 30.5% |
|  |  | % of Total | 5.2% | 8.5% | 11.7% | 5.2% | 0.0% | 30.5% |
| Total | | % within Q8 | 27.7% | 28.6% | 31.9% | 9.9% | 1.9% | 100.0% |
|  |  | % within Q21 | 100.0% | 100.0% | 100.0% | 100.0% | 100.0% | 100.0% |
|  |  | % of Total | 27.7% | 28.6% | 31.9% | 9.9% | 1.9% | 100.0% |

**Table 18: Result of Chi-square (𝜒²) Tests**

| **Test** | **Value** | **df** | **Asymp. Sig. (2-sided)** |
| --- | --- | --- | --- |
| Pearson Chi-square (𝜒²) | 14.608 | 8 | 0.067 |

**Table 19: Cross tabulation**

|  | | | Q29 | | | |  |  |
| --- | --- | --- | --- | --- | --- | --- | --- | --- |
|  |  |  | Strongly Disagree | Disagree | Neutral | Agree | Strongly Agree | Total |
| Q8 | Bank Employees | % within Q8 | 33.3% | 41.7% | 25.0% | 0.0% | 0.0% | 100.0% |
|  |  | % within Q29^[[13]](#footnote-13)^ | 9.1% | 8.6% | 5.2% | 0.0% | 0.0% | 5.6% |
|  |  | % of Total | 1.9% | 2.3% | 1.4% | 0.0% | 0.0% | 5.6% |
|  | Employees of corporations | % within Q8 | 22.8% | 26.5% | 29.4% | 14.7% | 6.6% | 100.0% |
|  |  | % within Q29 | 70.5% | 62.1% | 69.0% | 58.8% | 47.4% | 63.8% |
|  |  | % of Total | 14.6% | 16.9% | 18.8% | 9.4% | 4.2% | 63.8% |
|  | Lawyers | % within Q8 | 13.8% | 26.2% | 23.1% | 21.5% | 15.4% | 100.0% |
|  |  | % within Q29 | 20.5% | 29.3% | 25.9% | 41.2% | 52.6% | 30.5% |
|  |  | % of Total | 4.2% | 8.0% | 7.0% | 6.6% | 4.7% | 30.5% |
| Total | | % within Q8 | 20.7% | 27.2% | 27.2% | 16.0% | 8.9% | 100.0% |
|  |  | % within Q29 | 100.0% | 100.0% | 100.0% | 100.0% | 100.0% | 100.0% |
|  |  | % of Total | 20.7% | 27.2% | 27.2% | 16.0% | 8.9% | 100.0% |

**Table 20: Result of Chi-square (𝜒²) Tests**

| **Test** | **Value** | **df** | **Asymp. Sig. (2-sided)** |
| --- | --- | --- | --- |
| Pearson Chi-square (𝜒²) | 12.580 | 8 | 0.12 |

1. Question: You identify yourself as: 1. Right to Erase, 2. Right to Access, 3. Right to Erasure. [↑](#footnote-ref-1)
2. Indicate which rights you are aware of under the DPDP Act, 2023

   Section 11: Right to access information about personal data.

   Section 12: Right to correction and erasure of personal data

   Right to access

   Right to correction

   Right to erase

   All of the above [↑](#footnote-ref-2)
3. Q35: Your corporation provided information on the DPDP Act, 2023. [↑](#footnote-ref-3)
4. *Supra* note 2 [↑](#footnote-ref-4)
5. Identify the most important challenge you foresee in the implementation of the DPDP Act,2023

   Technical challenges

   Financial costs

   Staff training and awareness

   Changes to existing business processes

   Compliance monitoring and reporting [↑](#footnote-ref-5)
6. Your corporation is taking measures to comply with the DPDP Act, 2023? [↑](#footnote-ref-6)
7. The DPDP Act, 2023 is an easy to comply law for corporations [↑](#footnote-ref-7)
8. The DPDP Act, 2023 balances data protection with the need for data processing inbusiness operations [↑](#footnote-ref-8)
9. The DPDP Act, 2023 facilitates the Ease of Doing Business in India. [↑](#footnote-ref-9)
10. The DPDP Act 2023 fosters innovation. [↑](#footnote-ref-10)
11. The belief that the DPDP Act provides an effective grievance redressal mechanism is independent of professional association [↑](#footnote-ref-11)
12. The DPDP Act 2023 will be effective in addressing my concerns regarding data privacy [↑](#footnote-ref-12)
13. By repealing Sec 43A of the Information Technology Act,2000 the DPDP Act, 2023 doesaway with the distinction between ‘sensitive personal data’ and ‘personal data’. It isjustified. [↑](#footnote-ref-13)
