# Supplementary material for: Stakeholder perceptions of India’s Digital Personal Data Protection Act of 2023: an empirical study across legal, banking, and corporate sectors
Source: Front Sociol. 2026 May 29;11:1753383. doi: 10.3389/fsoc.2026.1753383 (PMC13259712; doi:10.3389/fsoc.2026.1753383)
Supplement: Supplementary file 2 [file Table_1.docx]

**Table 01: Scores of Independent Samples t-test of Awareness Levels**

| **Group** | **N** | **Mean** | **Std. Dev** | **Std. Error Mean** | **t** | **p** | **Cohen’s D** |
| --- | --- | --- | --- | --- | --- | --- | --- |
| Professionals | 213 | 28.72 | 4.67 | 0.32 | 3.39 | 0.001 | 0.348 |
| Laypersons | 167 | 27.19 | 4.10 | 0.31 |  |  |  |
